# Pharmacokinetics, safety and efficacy of an optimized dose of artemether–lumefantrine in the treatment of acute uncomplicated *Plasmodium falciparum* malaria in neonates and infants of less than 5 kg body weight: a multicentre, open-label, single-arm phase 2/3 study (CALINA)

**DOI:** 10.1186/s41182-025-00828-z

**Published:** 2025-11-06

**Authors:** Gildas Wounounou, Alfred B. Tiono, Bernhards Ogutu, Christine Manyando, Issaka Sagara, Stefan Schneitter, Quique Bassat, Myriam El Gaaloul, Anne Claire Marrast, Ivan Demin, Cornelis Winnips, Celine Risterucci, Sophie Hugot, Georg Hofstetter, Zhiyan Qian, Guoqin Su, Jie Zhang, Katalin Csermak Renner, Marc Cousin, Vinay Kumar Venishetty, Sarfaraz Sayyed, Preetam Gandhi, Berenger Kabore, The CALINA study group

**Affiliations:** 1 Saint Luc Hospital, Kisantu, Central Kongo, Democratic Republic of the Congo; 2 Groupe de Recherche Action en Santé (GRAS), Ouagadougou, Burkina Faso; 3 Kenya Medical Research Institute, Nairobi, Kenya; 4 CREATES, Strathmore University, Nairobi, Kenya; 5 National Health Research and Training Institute (Formerly TDRC), Ndola, Zambia; 6 University of Sciences, Techniques and Technologies of Bamako, Bamako, Mali; 7 Swiss Tropical and Public Health Institute, Allschwil, Switzerland; 8 University of Basel, Basel, Switzerland; 9 ISGlobal, Hospital Clínic–Universitat de Barcelona, Barcelona, Spain; 10 Centro de Investigação Em Saúde de Manhiça (CISM), Maputo, Mozambique; 11 ICREA, Pg. Lluís Companys 23, 08010 Barcelona, Spain; 12 Institut Clínic de Medicina I Dermatologia’ Hospital Clínic de Barcelona, Barcelona, Spain; 13 Facultat de Medicina I Ciències de La Salut, Universitat de Barcelona (UB), Barcelona, Spain; 14 CIBER de Epidemiología y Salud Pública, Instituto de Salud Carlos III, Madrid, Spain; 15 Medicines for Malaria Venture, Meyrin, Switzerland; 16 Novartis Pharma AG, Basel, Switzerland; 17 Novartis Pharmaceutical Corporation, East Hanover, NJ USA; 18 Novartis Healthcare Private Limited, Hyderabad, India; 19 Institut de Recherche en Sciences de La Sante, Clinical Research Unit of Nanoro (IRSS-CRUN), Ouagadougou, Burkina Faso

**Keywords:** *Plasmodium falciparum* malaria, Neonatal malaria, Artemether–lumefantrine, Infants, Efficacy, Safety, Pharmacokinetics, Bayesian borrowing analysis, Pharmacokinetic bridging

## Abstract

**Background:**

Treatment recommendations for malaria in infants of < 5 kg body weight (BW) are not evidence-based. Due to pharmacokinetic characteristics of this population, weight-based dose adjustments for antimalarials may be suboptimal. The 20 mg artemether:120 mg lumefantrine dispersible tablet, even with dose adjustment, may lead to artemether over-exposure and reduced lumefantrine exposure in patients < 5 kg. PBPK modelling predicted that a 1:12 artemether:lumefantrine ratio dispersible tablet should match efficacious and safe drug exposures in patients 5- < 15 kg treated with the current artemether–lumefantrine dispersible tablet: the CALINA study used an exposure-matching approach to confirm that drug exposures were comparable.

**Methods:**

Sequential age cohorts (Cohort 1: > 28 days; Cohort 2: 1–28 days) of patients < 5 kg with *Plasmodium falciparum* malaria received the new artemether–lumefantrine dispersible tablet (each dose 5 mg artemether: 60 mg lumefantrine) twice daily for 3 days. Artemether C_max_, and lumefantrine C_168h_ and C_max_ were compared with historical data from patients 5– < 15 kg treated with the current artemether–lumefantrine dispersible tablet. The primary endpoint was met if the 90% CI for artemether C_max_ contained the LS mean value from historical data (101 ng/mL). PCR-corrected and uncorrected ACPR at Days 15, 29 and 43 and parasite clearance time were evaluated. Adverse events, laboratory evaluations, and developmental assessments were performed.

**Results:**

In Cohort 1 (*N* = 22), geometric mean artemether C_max_ was 68.0 ng/mL (90% CI 45.1,103 ng/mL); therefore, C_max_ was comparable to that in historical data, meeting the primary endpoint. In Cohort 2 (*N* = 6), there were too few patients for formal analysis, but geometric mean artemether C_max_ was comparable to that in Cohort 1 (62.2 ng/mL, 90% CI 33.6,115 ng/mL). In both cohorts, lumefantrine C_168h_ and C_max_ were comparable to historical data. PCR-corrected Day 29 ACPR was 95.5% and 100% in Cohorts 1 and 2, respectively. Treatment was well-tolerated. Developmental assessments at 12 months of age were within the normal range.

**Conclusions:**

The optimized dose of artemether–lumefantrine (5 mg/60 mg) achieves the exposures required for optimal efficacy and safety in patients < 5 kg body weight with *P. falciparum* malaria, consistent with those in patients 5– < 15 kg treated with the current dispersible tablet (20 mg/120 mg).

*Trial registry*: Clinicaltrials.gov: NCT04300309.

**Supplementary Information:**

The online version contains supplementary material available at 10.1186/s41182-025-00828-z.

## Background

Children under 5 years of age, particularly in sub-Saharan Africa, are the population most at risk of death from malaria [1]. Young children are at high risk of both infection and death from malaria because they have yet to acquire immunity to *Plasmodium* species. Malaria in neonates and infants < 12 months of age, particularly in very young infants, is not well characterized. Even in malaria-endemic areas, infants below the age of 3 months or under 5 kg body weight are perceived to be protected from malaria, and misdiagnosis and mismanagement of the disease in this age group may occur as a result [2, 3]. Malaria in neonates can occur as congenital infection transferred from an infected mother, or may be acquired from a mosquito bite. Malaria in neonates up to 7 days of age is considered, by consensus, to be congenital malaria, given that it is believed that the incubation period after an infected mosquito bite is at least 7 days [3]. Data on the prevalence of malaria in patients below the age of 3 months or under 5 kg body weight are sparse. In a meta-analysis of data from endemic countries, congenital malaria as detected by microscopy was estimated to be present in approximately 34 per 1000 infants, with neonatal malaria (in the first 28 days of life) in 12 per 1000, with considerable heterogeneity between the studies included [2]. Another report found a prevalence of malaria in infants 0–6 months of age, based on PCR analysis, ranging between 3.7 and 22%, with the likelihood of infection increasing with age [4]. Infection in the youngest infants is often asymptomatic, but in some cases may lead to anaemia [5]. Malaria in neonates and young infants in endemic regions should therefore be considered as a differential diagnosis in cases of fever or non-specific symptoms, and may be consequential for patients. This indicates a need both for increased efforts to prevent neonatal malaria, via treatment and prevention of infection in pregnant women, and for the development of therapeutic guidelines and treatment options specific to very young infants [2].

Most clinical studies of antimalarials in paediatric patients exclude infants of < 6 months of age or < 5 kg body weight. At the time of writing, there are no approved malaria treatments for patients with a body weight < 5 kg, with the exception of artesunate/amodiaquine, which is approved for patients > 4.5 kg. Current WHO guidelines for patients < 5 kg body weight recommend treatment with artemisinin-based combination therapy (ACT) at the same mg/kg body weight target dose as for children weighing ≥ 5 kg with consideration of the safety and tolerability of the partner drug, but acknowledge that this recommendation is not evidence-based [6]. National guidelines for treatment of malaria in this age group vary widely [7–15].

Simple age- or weight-based adjustment of antimalarial doses based on those recommended for older children is not optimal for young infants, as these patients have distinct pharmacokinetic profiles compared with older patients, notably larger volumes of distribution, higher clearance rates, and immature enzyme systems [16]. Treatment needs for patients < 6 months of age or weighing < 5 kg therefore cannot be extrapolated accurately from older children or adults [17]. The absence of age- and weight-appropriate formulations of ACTs in this specific patient group, in addition to resulting in over- or under-dosing of ACT components, may also lead to use of off-label monotherapy, for example quinine or injectable artesunate [16, 18, 19], potentially posing a safety risk. There is therefore a clear unmet need for an age-appropriate formulation and dosing recommendation for antimalarial treatment in patients of < 5 kg body weight.

The artemether–lumefantrine dispersible tablet (20 mg artemether and 120 mg lumefantrine; Coartem^®^ Dispersible, Novartis, Basel) was initially developed for paediatric patients (body weight 5– < 35 kg, under 12 years of age). It was designed to be easier to administer than crushed tablets and was demonstrated to have similar efficacy, safety and pharmacokinetics to crushed tablets [20, 21]. In a study in which patients > 28 days old and < 5 kg body weight with uncomplicated falciparum malaria received artemether–lumefantrine dispersible tablet at the same dosage as approved for patients of body weight 5– < 15 kg (one dispersible tablet twice daily for three days), the artemether and dihydroartemisinin (DHA) systemic exposures were two- to three-fold greater than those seen in infants and children ≥ 5 kg. This study was terminated after completion of the first cohort (aged > 28 days) due to the higher than expected artemether exposure, with exposures being anticipated to be even higher in neonates. Lumefantrine exposure was similar to that in patients ≥ 5 kg, except at 7 days when mean lumefantrine concentration (C_168h_) was almost twofold greater in patients < 5 kg [22].

Tiono et al. [22] note that using, for example, one dispersible tablet once daily, rather than twice daily, for three days would not reduce the risk of artemether and DHA toxicity (as C_max_ values would remain elevated), and the decreased lumefantrine exposure might increase the risk of treatment failure. The difficulties of utilizing the existing dispersible tablet in very young infants led to the development of the paediatric formulation with an optimized dose and artemether:lumefantrine ratio for patients < 5 kg, with each dispersible tablet containing 2.5 mg artemether and 30 mg lumefantrine (i.e. an artemether:lumefantrine ratio of 1:12 rather than the 1:6 ratio in the existing dispersible tablet). When administered in patients < 5 kg body weight, at a dose of 5 mg artemether and 60 mg lumefantrine (i.e. two tablets), this ratio of components was predicted, based on physiologically based pharmacokinetic (PBPK) modelling [23], to match the observed artemether and lumefantrine exposures previously reported in patients 5– < 15 kg treated with the artemether–lumefantrine dispersible tablet [20, 21].

Here we describe the pharmacokinetics, tolerability, safety and efficacy of this fixed-ratio formulation of artemether–lumefantrine, specifically designed for patients weighing < 5 kg, in the treatment of uncomplicated *P. falciparum* malaria in infants and neonates of < 5 kg body weight, as characterized in a Phase II/III study (CALINA; NCT04300309). In view of the difficulties of conducting clinical trials in this patient population, given that infection is often asymptomatic or undetected, a pharmacokinetic bridging approach was used, with key PK parameters being compared with historical data from a previous study with the artemether–lumefantrine dispersible tablet in older children (NCT00386763 [20, 21]), with additional physiologically based PK (PBPK) modelling analysis (reported separately [24]).

## Methods

### Objectives

The primary objective of this study was to assess the key pharmacokinetic parameter of artemether (C_max_) in infants and neonates < 5 kg body weight dosed with the new formulation of artemether–lumefantrine dispersible tablet. The primary objective was considered to have been met if the 90% CIs for artemether C_max_ contained a predefined target value based on historical data, specifically those from children 5– < 15 kg body weight in a previous clinical trial (NCT00386763 [20, 21]). Secondary objectives were to assess other key pharmacokinetic parameters of artemether, dihydroartemisinin (DHA), and lumefantrine; to assess the safety and tolerability of the new formulation of artemether–lumefantrine dispersible tablet; and to assess efficacy in neonates and infants < 5 kg body weight.

### Overall design

**Fig. 1:**
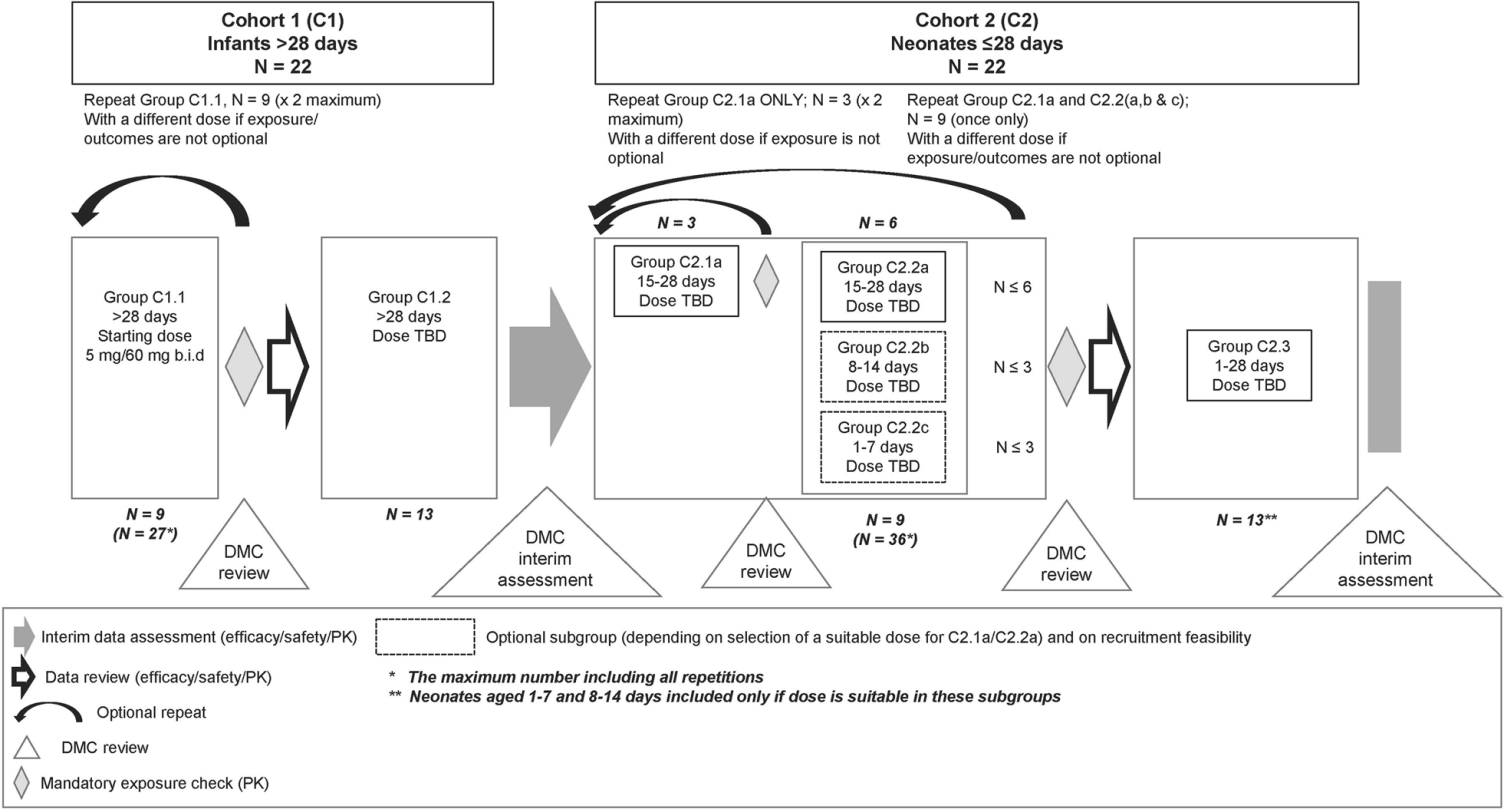
Title: Study design

**Fig. 2:**
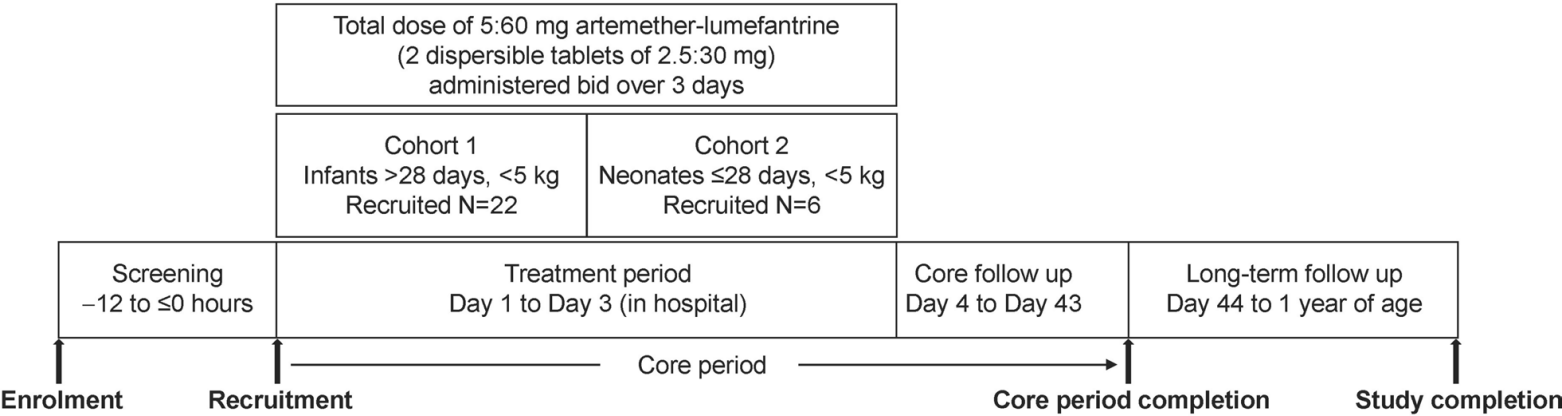
Title: Patient participation

CALINA was a Phase II/III multicentre, open-label, single-arm, sequential-cohort study, with adaptive design (Fig. 1). Participants were treated in an inpatient setting for the first 3 days and followed up as outpatients. The open-label, adaptive, sequential-group design study was chosen to minimize the risk to patients by studying first a cohort of infants (> 28 days of age, < 5 kg) similar to a population already evaluated previously (i.e. infants ≥ 5 kg body weight, irrespective of age [20, 21]), before recruiting patients ≤ 28 days of age as the second cohort. An independent data monitoring committee reviewed efficacy, safety and PK data prior to each key decision point (Fig. 1). Pharmacokinetic exposure checkpoints were 1) after the first nine patients in Cohort 1 had been dosed and followed up to Day 15 (which determined whether the next 13 patients were recruited for Cohort (1), and (2) after the first three patients in Cohort 2 had been dosed and followed up to Day 15; this determined whether the next six patients would be recruited to Cohort 2. A third checkpoint was planned after nine patients had been dosed and followed up in Cohort 2 but was not implemented in practice due to limited patient recruitment. It was planned to enrol and treat a maximum of 22 patients in each cohort. Per protocol, each cohort could be repeated using different doses, although this was not necessary in practice. The core study duration was 43 days. The first day of treatment was designated Day 1; treatment was given on Days 1–3, with follow-up from Days 4 to 43, with a long-term developmental follow-up assessment for each patient at 12 months of age (Fig. 2).

The study protocol was reviewed and approved by the institutional ethics committee or review board for each centre and by national health authorities. For the two centres that recruited patients in Burkina Faso, the protocol was approved by Comité d’Ethique pour la Recherche en Santé, Ministère de l’Enseignement Supérieur de la Recherche Scientifique et de l’innovation, Ouagadougou, Burkina Faso. The approving body for the centre in the Democratic Republic of the Congo was Comité National d’Ethique de la Santé, Ministère de la Santé Publique, Kinshasa, Democratic Republic of the Congo. Informed consent was obtained from the legally acceptable representative, i.e. parent or legal guardian, of each patient.

### Participants

Any patient attending neonatal or paediatric clinics at the study centres with consenting parents or legal guardians was tested for malaria by blood microscopy, as were patients whose mothers had a history of malaria during pregnancy, irrespective of the presence or absence of malaria symptoms. The study included infants and neonates < 5 kg body weight with acute uncomplicated *P. falciparum* malaria, which could be either congenital (more relevant to Cohort 2) or acquired, and symptomatic or asymptomatic. Patients could be of either sex, with body weight < 5 kg but ≥ 2 kg. Patients were recruited in two sequential age-descending cohorts: Cohort 1 included patients aged > 28 days with parasitaemia ≥ 500 to < 100,000 parasites/µL, and Cohort 2 included patients aged 1–28 days with ≥ 100 to < 100,000 parasites/µL.

Key exclusion criteria were: head circumference < − 2 standard deviations z-score in cm following WHO age and sex specific reference curves (i.e. suspicion of microcephaly); severe malaria (according to WHO 2015 definition [25]); in Cohort 1, patient’s or patient’s mother’s current treatment with antiretrovirals; in Cohort 2, mother’s known HIV-positive status at patient’s birth or mother’s current treatment with antiretrovirals; severe malnutrition; presence of the following signs of a critical condition: apnoea–bradycardia, sustained bradycardia, tachycardia, desaturation, hypotension, hypothermia, or other severely deteriorated general condition (based on IMCI criteria in sick infants; [26]). Full inclusion and exclusion criteria for the study can be found in Additional File 1.

### Interventions

Eligible patients received treatment with an artemether–lumefantrine paediatric formulation with an optimized dose for patients < 5 kg body weight. The first nine patients received two dispersible tablets (providing a 5 mg artemether: 60 mg lumefantrine dose) twice daily for three consecutive days. If this dose did not achieve an artemether C_max_ within approximately twofold the value known to be safe and efficacious in previous studies (101 ng/mL, please refer to the statistical methodology below), a further nine patients would be recruited and treated with a different dose. This process could be repeated if necessary. The tablets were completely dispersed using a syringe in a small amount (up to 3 mL) of clean water and were immediately administered to patients using the syringe; the syringe was then rinsed with water that was also administered to the patient. Wherever possible, treatment was preceded and/or followed by food or drinks rich in fat, such as breast milk or formula milk. The same dosing regimen and method of administration were used in both cohorts.

Blood samples were taken for determination of parasitaemia at baseline, then at 8, 24, 36, 48, 60, 72, 96 and 168 h (7 days), then at 14, 28 and 42 days after initiation of treatment. Blood samples for pharmacokinetic analysis were obtained according to two different schedules (1, 2, 62, 66 and 168 h after the first dose of study medication or 1, 2, 68, 84 and 168 h; patients were randomized 1:1 to these specific schedules); this meant that PK data were available at 1, 2, 62, 66, 68, 84 and 168 h. Samples for blood chemistry and haematology assessments were taken at baseline, and at 24, 60, and 168 h, and at 14 and 42 days after the first dose. The maximum volume of blood that could be sampled over the duration of the study was 2.2–2.3 mL/kg body weight (as measured at the screening visit).

### Outcomes

Pharmacokinetics: the primary endpoint of the study was the C_max_ of artemether, as represented by the highest concentration in blood samples collected at 1 h and 2 h following the first dose. Secondary pharmacokinetic endpoints were lumefantrine concentration at Day 8, i.e. 7 days after the start of treatment (C_168h_), and DHA and lumefantrine C_max_. Lumefantrine, artemether and DHA were measured in plasma using liquid chromatography tandem mass spectrometry methods. Lower limits of quantification were 50.0 ng/mL for lumefantrine and 3.0 ng/mL for artemether and DHA. Concentrations below the lower limit of quantification were treated as zero.

Efficacy was assessed in terms of PCR-corrected adequate clinical and parasitological response (ACPR) at Days 15, 29 and 43. ACPR was defined as clearance of initial parasitaemia and maintenance of blood slide negativity (no recrudescence in the case of PCR-corrected ACPR, no parasite reappearance for uncorrected ACPR) over the course of follow-up, together with the absence of malaria-related clinical signs and symptoms. Uncorrected ACPR was evaluated at Days 8, 15, 29 and 43. Other efficacy assessments were rates of recrudescence and new infection at Days 15, 29 and 43; parasite clearance time (PCT) and fever clearance time (FCT). Efficacy assessments were based on parasitaemia determinations in peripheral blood and body temperature measurement, in addition to signs and symptoms of early treatment failure (ETF). Parasitaemia was determined by examination of Giemsa-stained thick and thin films. Validated microscopy examination methods were used to quantify the malaria asexual parasites and gametocytes in blood samples [27, 28]; parasite counts from local laboratories were used for study inclusion, and central laboratory readings were used, including on baseline samples, to ensure standardiation of data for efficacy analyses.

Safety was assessed in terms of adverse events (AEs), including AEs of special interest (AESIs), serious adverse events (SAEs), and clinical laboratory assessments (clinical chemistry and haematology). AESIs were defined as AEs related to QT prolongation, haemolysis, haemoglobinuria, anaemia and blackwater fever, and hepatotoxicity/hepatic disorder. Vital signs and weight and height data were also recorded. Neurodevelopmental assessments were conducted during the study and at the follow-up visit when each patient was 12 months old, using the Shoklo Malaria Research Unit (SMRU) assessment [29, 30]. Head circumference was also measured at 12 months of age for each patient, for comparison with WHO reference ranges [31].

### Statistical methodology

The sample size for this study was calculated and justified based on the precision of estimating artemether C_max_ (primary endpoint) and lumefantrine C_168h_ (secondary endpoint) separately for Cohort 1 and Cohort 2, assuming that the standard deviation of log values was the same as that in a previous study using artemether–lumefantrine dispersible tablet in patients of < 5 kg body weight (EudraCT 2011-005852-33 [22]). The sample size included 10% more patients to account for dropouts without evaluable PK data. The study planned to enrol and treat an approximate sample size of 44 patients (approximately 22 patients in each Cohort). If exposure was found to be inadequate at the predefined PK checkpoints, additional patients could be recruited up to a maximum of 98 patients overall.

The PK analysis set, defined as all patients that received any study treatment, had *P. falciparum* present at the screening visit and had evaluable PK parameter data that were used for the analysis of artemether C_max_ after the first dose: 90% 2-sided confidence intervals for the geometric mean were presented by cohort. Calculation was based on the log-normal distribution. The primary objective was considered to have been met if the 90% CIs for artemether C_max_ contained a predefined target value of 101 ng/mL. This value is based on historical data, specifically those from children 5– < 15 kg body weight in a previous clinical trial (NCT00386763: the efficacy and safety results from this study were previously published [20], as were pharmacokinetic data [21]). As that publication [21] did not report lumefantrine C_168h_, and other parameters were reported as arithmetic means rather than geometric means (as is currently standard practice when reporting pharmacokinetic data), individual patient data from patients of 5 < 15 kg body weight treated with the artemether–lumefantrine dispersible tablet in Study NCT00386763 were re-analysed to calculate medians and geometric means for artemether and DHA C_max_, and lumefantrine C_max_ and C_168h_. Target values for these parameters in the current study were based on this analysis [Additional File 2].

For lumefantrine C_168h_, 90% 2-sided CIs of geometric mean were calculated, based on the log-normal distribution by cohort and treatment group using the PK set. Lumefantrine C_168h_ was considered to be comparable with historical data if the upper limit of 90% CIs is not less than the historical values (i.e. 212 ng/mL from 5 to < 15 kg body weight in Study NCT00386763). A similar approach to that for artemether C_max_ was used for lumefantrine C_max_ (reference value 3900 ng/mL) and DHA C_max_ (reference value 31.7 ng/mL).

PCR-corrected and uncorrected ACPR rates at Days 15, 29 and 43 were calculated with 95% 2-sided CIs using the Clopper–Pearson method for each cohort using the full analysis set (FAS), defined as all patients who received any study treatment and had *P. falciparum* present at the screening visit. For PCR-corrected ACPR rates, the same analysis was also performed using the per protocol set (PPS), defined as patients in the FAS who did not have important protocol deviations affecting efficacy, took at least 80% of study medication, and for whom PCR-corrected cure status at Day 29 could be defined.

Rates of recrudescence and new infection at Days 15, 29 and 43 were estimated using the Kaplan–Meier method based on the subset of the FAS who had clearance of initial infection by Day 7. Time to event (recrudescence or new infection) was calculated from the time of first study medication to the date of first event if a patient experienced the event and was censored at the time of last parasite assessment if a patient did not experience the event. Patients with new infection were censored at the time of first new infection when analysing time to recrudescence, and patients with recrudescence were censored at the time of first recrudescence when analysing time to new infection. Undetermined treatment failures due to missing PCR data were considered as censored at the time of treatment failure.

For PCT and FCT, descriptive statistics were presented for each treatment by cohort using the Kaplan–Meier method based on the FAS. Kaplan–Meier curves were provided. If a patient received rescue medication before parasite or fever clearance, the time to event was censored at the first use of rescue medication.

As the study was not powered to derive conclusions on the PCR-corrected ACPR at Day 29 meeting a specific efficacy threshold, a Bayesian borrowing approach was used to reanalyse the PCR-corrected ACPR at Day 29 in the context of previous studies with artemether–lumefantrine. Historical data from studies conducted by the sponsor with artemether–lumefantrine were synthesized using a Bayesian random effects meta-analysis. The objectives of this analysis were to confirm that the PCR-corrected ACPR at Day 29 in the current study was in alignment with historical data and to provide a more precise estimate of PCR-corrected ACPR at Day 29. PCR-corrected ACPR at Day 29 was calculated by varying the amount of borrowing from historical data for Cohort 1 alone, Cohort 2 alone, and Cohorts 1 and 2 pooled, each using 2 different analysis sets (PPS and FAS). Further details of the Bayesian borrowing analysis methodology can be found in Additional File 3.

Safety was assessed using the Safety Analysis Set (included all patients who received at least one dose of study treatment). Adverse events were summarized by cohort, by MedDRA system organ class (SOC) and preferred term. AESIs were prospectively defined as AEs related to QT prolongation, haemolysis, haemoglobinuria, anaemia and black water fever, and hepatotoxicity/hepatic disorders.

## Results

### Patients

The study was conducted between December 21st, 2020 (first patient first visit) and May 10th, 2024 (last patient last visit, i.e. safety follow-up at 12 months of age). The core part of the study was terminated after completion of Cohort 1 and enrolment of six patients into Cohort 2; termination was due to difficulty of recruitment into Cohort 2 and was not due to any safety concerns or lack of efficacy. Patients were screened at four centres in Burkina Faso, the Democratic Republic of the Congo, and Mali, and were enrolled and treated at two sites in Burkina Faso and one site in the Democratic Republic of the Congo. In total, 69 patients were screened and 28 were enrolled: of these, 22 were in Cohort 1 and six in Cohort 2. All patients completed treatment and the core follow-up period (up to Day 43). Long-term follow-up (when patients reached 12 months of age) was completed by 27/28 (96.4%) patients – one patient in Cohort 1 was lost to follow-up. All patients in both cohorts were included in the FAS, Safety Analysis Set, and PK analysis set. In Cohort 1, 17/22 (77.3%) patients were included in the Per Protocol set, as were all patients in Cohort 2. All patients in Cohorts 1 and 2 received artemether–lumefantrine at the 5 mg/60 mg dose; no dose adjustments were required.

Demographic characteristics are summarized for the FAS in Table 1. Overall, 18/28 (64%) patients were female. Median age in Cohort 1 was 96 days; in Cohort 2 median age was 23 days; five of the six patients were aged 15–28 days and the remaining patient was 1 day old.

**Table 1:** Demographic characteristics (FAS)

Characteristic	Cohort 1	Cohort 2	All patients
	*N* = 22	*N* = 6	*N* = 28
Age (days)			
*n*	22	6	28
Mean (SD)	96.5 (28.40)	19.5 (9.22)	80.0 (40.95)
Median (range)	96.0 (53.0–157.0)	22.5 (1.0–26.0)	83.0 (1.0–157.0)
Sex—*n* (%)			
Male	7 (31.8)	3 (50.0)	10 (35.7)
Female	15 (68.2)	3 (50.0)	18 (64.3)
Height (cm)			
*n*	22	6	28
Mean (SD)	57.0 (2.73)	52.1 (1.86)	55.9 (3.25)
Median (range)	57.8 (52.0–61.0)	51.5 (50.0–55.0)	55.3 (50.0–61.0)
Birth weight (kg)			
*n*	20	6	26
Mean (SD)	2.82 (0.620)	3.10 (0.456)	2.88 (0.590)
Median (range)	2.73 (2.00–4.50)	2.95 (2.60–3.80)	2.80 (2.00–4.50)
Weight (kg)			
*n*	22	6	28
Mean (SD)	4.68 (0.337)	3.58 (0.512)	4.44 (0.591)
Median (range)	4.82 (3.89–4.98)	3.50 (2.80–4.24)	4.76 (2.80–4.98)
Head circumference (cm)			
*n*	22	6	28
Mean (SD)	39.0 (1.65)	36.2 (1.83)	38.4 (2.04)
Median (range)	39.0 (36.0–42.0)	36.5 (34.0–38.0)	38.0 (34.0–42.0)

Age is calculated at the time of screening; weight and height baseline values are used

Baseline malaria characteristics are summarized in Table 2.

**Table 2:** Malaria characteristics at baseline (FAS)

Characteristic	Cohort 1	Cohort 2	All patients
	*N* = 22	*N* = 6	*N* = 28
Body temperature (°C)			
*n*	22	6	28
Mean (SD)	37.0 (0.97)	37.1 (0.45)	37.0 (0.88)
Median (range)	36.7 (36.0–39.4)	36.9 (36.6–37.8)	36.8 (36.0–39.4)
Body temperature (axillary) category (°C)–*n* (%)			
< 37.5	18 (81.8)	5 (83.3)	23 (82.1)
37.5–< 39	2 (9.1)	1 (16.7)	3 (10.7)
≥ 39	2 (9.1)	0	2 (7.1)
Parasite counts (central laboratory)			
*Plasmodium* species–*n* (%)			
*P. falciparum* asexual forms	21 (95.5)	6 (100)	27 (96.4)
*P. falciparum* gametocytes	4 (18.2)	1 (16.7)	5 (17.9)
*P. malariae*	1 (4.5)	0	1 (3.6)
*P. falciparum *density (/µL)			
*n*	21	6	27
Mean (SD)	31,500 (45,300)	11,400 (20,400)	27,000 (41,600)
Geometric mean	10,200	2800	7700
Median (range)	8400 (748–156,400)	3660 (384–52,700)	7020 (384–156,400)
Categories – n (%)			
< 500	0	1 (16.7)	1 (3.6)
500–< 2,000	4 (18.2)	2 (33.3)	6 (21.4)
2,000–< 5,000	4 (18.2)	0	4 (14.3)
5,000–< 15,000	5 (22.7)	2 (33.3)	7 (25.0)
15,000–< 50,000	4 (18.2)	0	4 (14.3)
≥ 50,000	4 (18.2)	1 (16.7)	5 (17.9)

Baseline is defined as the last available assessment/measurement prior to first administration of study treatment

Malaria blood smear readings from central reference laboratory are used

Central laboratory microscopy showed 27/28 (96.4%) of patients were positive for *P. falciparum* asexual forms at baseline; the other patient had a missing central laboratory sample, but the local laboratory sample confirmed positivity for this patient. One patient had a central laboratory asexual form count of 156,400/µL, which was outside the entry criteria for the study; however, the local laboratory count (which was used for purposes of study inclusion) for this patient was < 100,000/µL, and the patient was included in the study. Gametocytes were present in five patients (17.9%). One patient had *P. malariae* co-infection. The majority of patients (23/28, 82.1%) did not have fever (baseline body temperature < 37.5 °C) at baseline; this was not unexpected as fever was not an entry criterion for the study, given that malaria in patients in this age group may have atypical symptoms or be asymptomatic [5].

### Pharmacokinetics

**Fig. 3:**
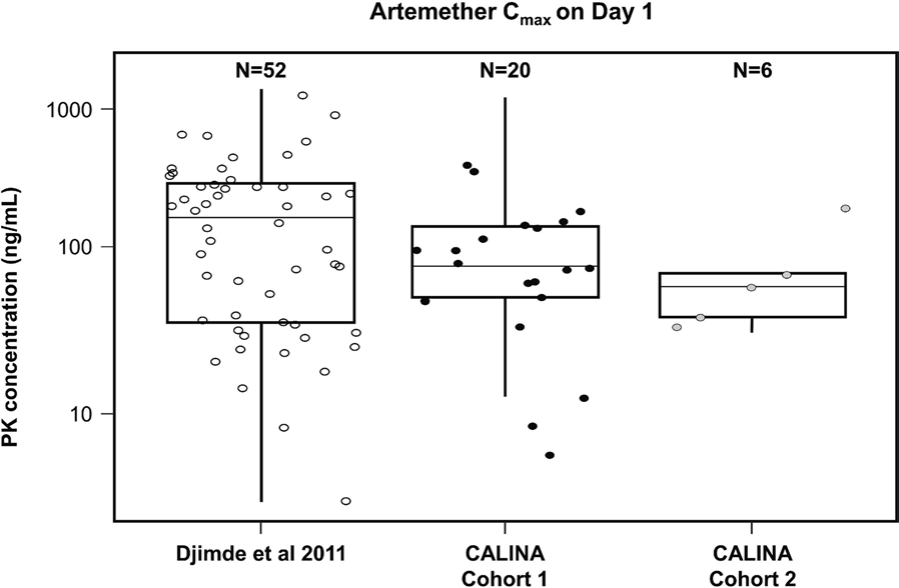
Title: Artemether C_max_ on Day 1, Cohorts 1 and 2, and historical data. Legend: the lower, middle and upper hinges of the box correspond to the first, second and third quartiles, i.e. 25th, 50th and 75th percentiles, respectively. The upper whisker extends from the hinge to the largest value no further than 1.5 times the interquartile range; the lower whisker extends from the hinge to the smallest value at least 1.5 times the interquartile range. Data from Study NCT00386763 [20, 21] are from patients of body weight 5– < 15 kg treated with the artemether–lumefantrine dispersible tablet

The primary endpoint for Cohort 1, artemether C_max_ after the first dose, is summarized for the PK analysis set in Table 3. The 90% CIs for artemether C_max_ (45.1–103 ng/mL) contained the predefined target geometric mean value, based on historical data, of 101 ng/mL. The primary objective of the study was therefore considered to have been met, as artemether C_max_ was within the safe and efficacious range as defined in older paediatric patients. The sample size in Cohort 2 was too small for formal statistical interpretation, but the artemether C_max_ geometric mean and 90% CIs were consistent with those in Cohort 1, within the targeted range (the 90% CIs contained the 101 ng/mL value). Figure 3 shows a box and whisker plot and individual values of artemether C_max_ for the study NCT00386763 [20, 21] and both cohorts in the current study.

**Table 3:** Summary of artemether C_max_ after first dose (PK Set)

Statistics	Cohort 1 *N* = 22	Cohort 2 *N* = 6
C_max_ (ng/mL)		
*n*^a^	20	5
Mean (SD)	102 (84.6)	75.0 (57.3)
CV% mean	83.3	76.3
Median (range)	79.0 (5.77–321)	58.4 (33.9–174)
Geo-mean (90% CI)	68.0 (45.1–103)	62.2 (33.6–115)

*n*: number of patients with evaluable PK parameters (Cohort 1: > 28 days and Cohort 2: 15–28 days)

Geo-mean: geometric mean

CV% = coefficient of variation (in percent) = SD/mean*100

CV% geo-mean = (sqrt(exp(variance of log-transformed data)—1))*100

Artemether C_max_ represents higher concentration between the two concentration time points (at around 1 h and 2 h) after first dose

^a^ Two patients were excluded from analysis due to sample identification issues in Cohort 1 and PK samples were not available for one patient in Cohort 2

**Fig. 4:**
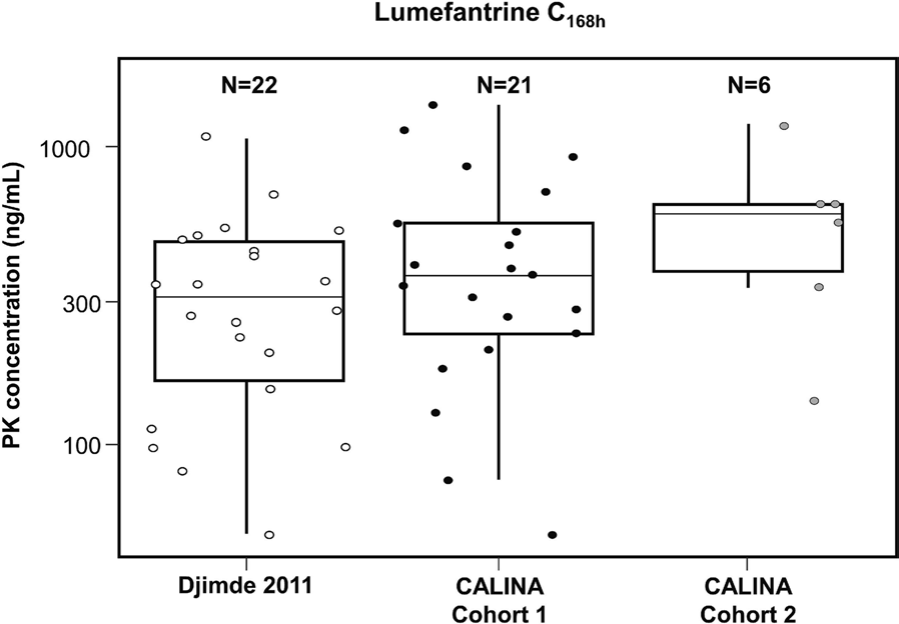
Title: Lumefantrine C_168h_, Cohorts 1 and 2 and historical data. Legend: the lower, middle and upper hinges of the box correspond to the first, second and third quartiles, i.e. 25th, 50th and 75th percentiles, respectively. The upper whisker extends from the hinge to the largest value no further than 1.5 times the interquartile range; the lower whisker extends from the hinge to the smallest value at least 1.5 times the interquartile range. Data from Study NCT00386763 [20, 21] are from patients of body weight 5– < 15 kg treated with the artemether–lumefantrine dispersible tablet

Table 4 summarizes secondary pharmacokinetic endpoints. Lumefantrine C_168h_ in Cohort 1 was also within the range observed in older paediatric patients; the upper limit of the 90% CIs (250–498 ng/mL) was greater than the targeted predefined value of 212 ng/mL. In Cohort 2, lumefantrine C_168h_ geometric mean and 90% CIs were consistent with those in Cohort 1. Figure 4 shows a box and whisker plot and individual values of lumefantrine C_168h_ for each cohort and for historic data.

**Table 4:** Summary of lumefantrine C_168h_ and C_max_, and DHA C_max_ (PK Set)

Statistic	Cohort 1 *N* = 22		Cohort 2 *N* = 6
Lumefantrine C_168h_ (ng/mL)			
*n*	22		6
Mean (SD)	488 (369)		578 (346)
CV% mean	75.5		59.8
Median (range)	378 (0–1370)		594 (140–1160)
Geo-mean (90% CI)	353 (250–498)		480 (265–870)
Lumefantrine C_max_ (ng/mL)			
*n*		22	6
Mean (SD)		3730 (2040)	4210 (2180)
CV% mean		54.6	51.8
Median (range)		3660 (596–9390)	4800 (865–7200)
Geo-mean (90% CI)		3180 (2530–4000)	3510 (1880–6540)
DHA C_max_ (ng/mL)			
*n*^a^		20	5
Mean (SD)		17.3 (14.5)	18.8 (13.6)
CV% mean		83.7	72.3
Median (range)		13.6 (0–55.6)	11.8 (8.40–41.4)
Geo-mean (90% CI)		11.5 (7.58–17.4)	15.7 (8.53–28.9)

*n*: number of patients with evaluable PK parameters (Cohort 1: > 28 days and Cohort 2: 1–28 days)

Geo-mean: geometric mean

CV% = coefficient of variation (in percent) = SD/mean*100

CV% geo-mean = (sqrt(exp(variance of log-transformed data)—1))*100. A value of 0 is imputed with LLOQ/2 when deriving the geometric mean and the corresponding confidence interval

For lumefantrine, C_max_ represents highest concentration among four concentration time points collected post last dose

For DHA: C_max_ represents higher concentration between the two concentration time points (at around 1 h and 2 h) after first dose

^a^ Two patients are excluded from analysis due to sample identification issues in Cohort 1 and PK samples were not available for one patient in Cohort 2

Similarly, lumefantrine C_max_ in both cohorts was consistent with values from historical data, with 90% CIs for the geometric means including the target value of 3900 ng/mL. For DHA C_max_ in both cohorts, the upper limits of the 90% CIs for the geometric mean were lower than the target value of 31.7 ng/mL. However, the study was not powered for comparison of DHA concentrations with those from historical data.

### Efficacy

For Cohort 1, the PCR-corrected ACPR in the FAS at Day 29 was 95.5% (21/22 patients). Five patients in Cohort 1 received prohibited concomitant medication (erythromycin, used to treat a variety of infections) and were excluded from the Per Protocol set, where the Day 29 PCR-corrected ACPR was 17/17 (100%). In Cohort 2, the Day 29 PCR-corrected ACPR was 6/6 (100%) in both the FAS and Per Protocol set, and at Days 15 and 43. One patient in Cohort 1 had a confirmed recrudescence at Day 29, and one patient had the reappearance of parasites at Day 43 but did not have a sample available for PCR analysis to confirm recrudescence or reinfection: this latter patient was categorized as a treatment failure and recrudescence.

The uncorrected ACPR at Day 29 in the FAS was 82.1% overall (23/28 patients); 77.3% (17/22) patients in Cohort 1 and 100% (6/6) in Cohort 2; in addition to the patient with recrudescence, four patients in Cohort 1 had reinfection by Day 29.

**Fig. 5:**
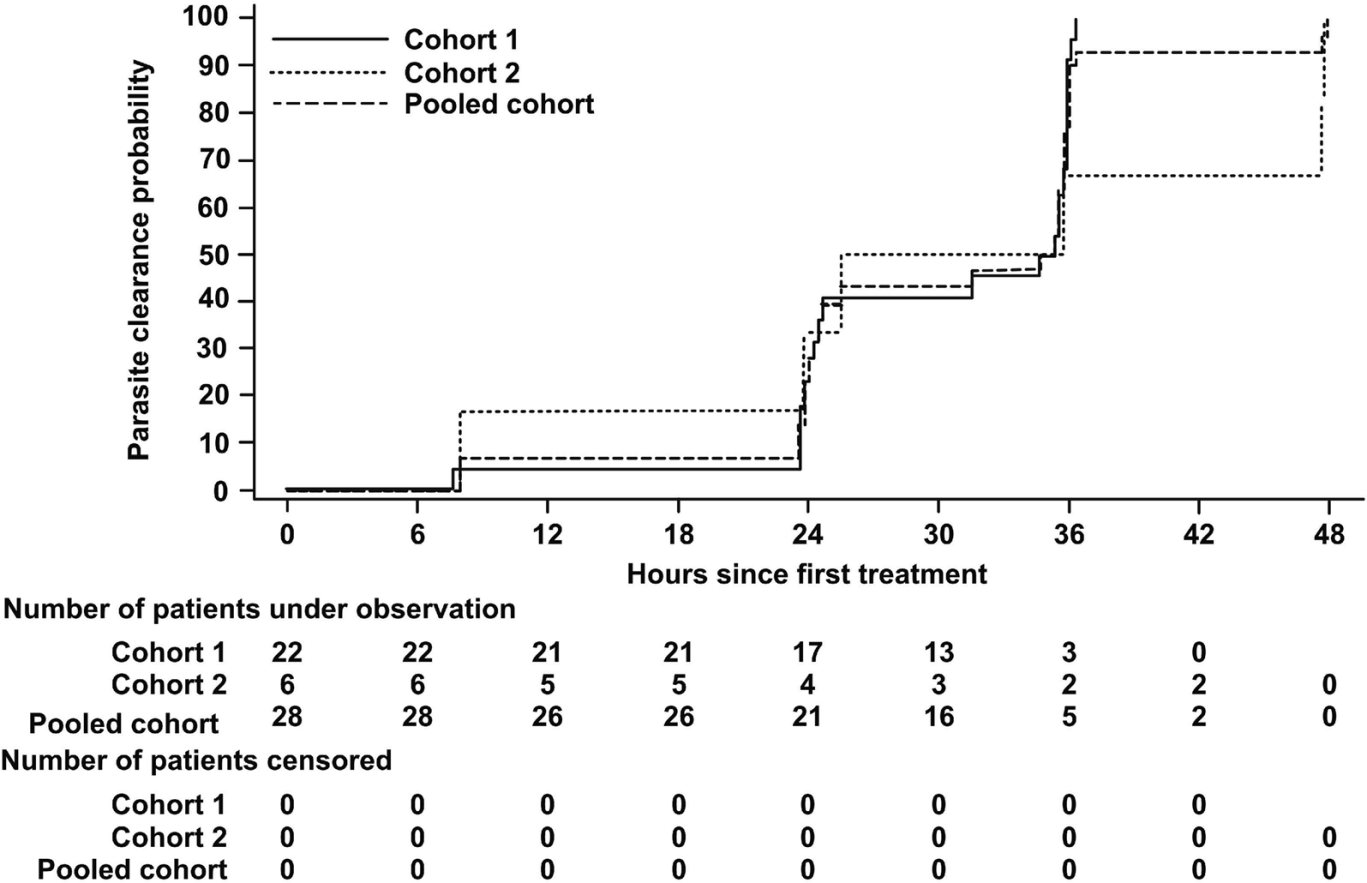
Title: Kaplan–Meier plot of parasite clearance time (FAS)

Figure 5 shows a Kaplan–Meier plot of PCT for the FAS, by cohort. Median PCT was 35 h overall (Cohorts 1 and 2 combined) and in Cohort 1, and 30.6 h in Cohort 2. For patients who were febrile at baseline, median FCT was 15.7 h in Cohort 1 (four patients) and 7.6 h in both Cohort 2 (one patient) and all patients (five patients).

Using a Bayesian borrowing approach, PCR-corrected ACPR at Day 29 observed in the current study was in alignment with PCR-corrected ACPR at Day 29 in historical studies regardless of cohort and analysis set, which confirms the relevance of historical data. For the pre-specified borrowing with a weight of 0.8 on the informative meta-analytic-predictive (MAP) prior from historical data, the posterior probability of response rate > 90% is greater than 0.975 regardless of cohort and analysis set. With the consistent results, the Cohorts 1 and 2 pooled FAS includes all patients and is considered the most relevant analysis set. For Cohorts 1 and 2 pooled FAS and the pre-specified borrowing with a weight of 0.8 on the informative MAP prior, the median response rate of the posterior distribution is 97.5% (95% credible interval [CrI]: 92.8–99.0) and the probability of a response rate > 90% is 0.993. Further, a sensitivity analysis identified that borrowing with a weight of 0.4 on the informative MAP prior leads to a median response rate of 97.5% (95% CrI: 90.3–99.3), a posterior probability of 0.977 that the response rate is greater than 90%, and a posterior probability of 0.875 that the response rate is greater than 95%.

### Tolerability and safety

No deaths, SAE, AEs leading to discontinuation, or prospectively specified AESIs were reported. Treatment was well-tolerated, and most AEs reported during the study could be explained by the underlying malaria; the most common AEs were malaria (i.e. recurrence of parasites), pyrexia, anaemia, and vomiting (Table 5). Most AEs were of moderate intensity; two patients had severe AEs—one patient with pyrexia (on Day 2, resolved on Day 3), the other with anaemia (observed on Day 2); neither event was related to study medication and both were probably due to malaria, in view of the timing. Both patients were in Cohort 1. Vomiting, on Day 1 in one patient in Cohort 2 and of mild intensity, was the only AE considered by the investigators to be related to study medication; other AEs of vomiting were not reported as treatment related. Overall, a total of eight AEs of vomiting following drug administration were reported, in seven patients (one patient in Cohort 1 reported two AEs of vomiting); all patients who vomited were successfully re-dosed within 1 h of the initial administration.

**Table 5:** Adverse events, regardless of treatment relationship, by preferred term and worst severity (safety analysis set)

Preferred term	Maximum severity	Cohort 1 *N* = 22 *n* (%)	Cohort 2 *N* = 6 *n* (%)	All patients *N* = 28 *n* (%)
Any adverse event	Mild	2 (9.1)	2 (33.3)	4 (14.3)
	Moderate	13 (59.1)	2 (33.3)	15 (53.6)
	Severe	2 (9.1)	0	2 (7.1)
Pyrexia	Mild	3 (13.6)	1 (16.7)	4 (14.3)
	Moderate	4 (18.2)	1 (16.7)	5 (17.9)
	Severe	1 (4.5)	0	1 (3.6)
Malaria	Mild	3 (13.6)	0	3 (10.7)
	Moderate	6 (27.3)	0	6 (21.4)
Anaemia	Moderate	6 (27.3)	1 (16.7)	7 (25.0)
	Severe	1 (4.5)	0	1 (3.6)
Vomiting	Mild	5 (22.7)	1 (16.7)	6 (21.4)
	Moderate	1 (4.5)	0	1 (3.6)
Bacterial rhinitis	Moderate	2 (9.1)	0	2 (7.1)
Abdominal pain	Mild	0	1 (16.7)	1 (3.6)
Blood magnesium increased	Mild	1 (4.5)	0	1 (3.6)
Dermatitis	Mild	1 (4.5)	0	1 (3.6)
Dermatitis infected	Moderate	1 (4.5)	0	1 (3.6)
Ear infection	Moderate	0	1 (16.7)	1 (3.6)
Gastrointestinal fungal infection	Mild	0	1 (16.7)	1 (3.6)
Influenza	Mild	1 (4.5)	0	1 (3.6)
Injection site phlebitis	Mild	1 (4.5)	0	1 (3.6)
Rhinitis	Mild	0	1 (16.7)	1 (3.6)

Preferred terms are sorted in descending frequency of AEs based on the overall count in the cohort

A patient with multiple occurrences of an AE is counted only once in the AE category

A patient with multiple AEs is counted only once in the total row

MedDRA Version 26.1 was used for the reporting of AEs

Most patients in Cohort 1 (21/22) had low haemoglobin (< 13 g/dL) at baseline; mean haemoglobin increased from baseline (8.95 g/dL) to Day 43 (10.11 g/dL)). In Cohort 2, mean haemoglobin level at baseline was 13.23 g/dL, which decreased by Day 43 (10.27 g/dL); three of six patients in Cohort 2 had low haemoglobin at baseline, but none required blood transfusion. There was no evidence of haemolysis in any patient in either cohort.

Four patients in Cohort 1 had abnormalities of liver function tests. One patient had elevated serum alanine aminotransferase (ALT) over three times the upper limit of normal (ULN) on Day 2 (127.7 U/L, increased from 84.8 U/L at baseline, normal range: 0–40 U/L), which normalized by Day 8 (29.9 U/L). Three patients had bilirubin increases to more than 1.5-fold ULN at Day 43; two of these patients had malaria AEs; one of the patients had elevated bilirubin at baseline. In Cohort 2, one patient had ALT of 119.3 U/L on Day 8, that increased from a normal baseline value (33 U/L), and remained elevated at Day 43 (51.7 U/L), together with an aspartate aminotransferase concentration of 120.4 U/L (normal range: 0–100 U/L), increased from 57.8 U/L at baseline, that decreased to 48.5 U/L at Day 43; baseline bilirubin was high in this patient but normalized by Day 43. No AEs related to liver function tests were reported, and none of these abnormalities were considered clinically significant by the investigators. The SMRU neurodevelopment assessment was performed at Day 4 and at the follow-up visit when patients reached 12 months of age. Results at 12 months of age are shown in Table 6. No adverse findings in the neurodevelopmental status for any of the parameters at 12 months of age were reported in 27 patients across Cohort 1 and Cohort 2 (one patient in Cohort 1 was lost to follow-up). Based on historical controls in published literature [30], a total score of < 52 identifies infants in need of attention or further investigation. In this study median total score was 93, and the minimum was 78.

**Table 6:** SMRU neurodevelopmental assessment by cohort at 12 months age (FAS)

Time point assessment	Cohort 1 *N* = 22	Cohort 2 *N* = 6	All patients *N* = 28
12 months of age			
Total scores for motor milestones (Section A)			
*n*	21	6	27
Mean (SD)	32.2 (2.84)	34.2 (3.13)	32.6 (2.96)
Median	33.0	35.0	34.0
Range	28–37	29–37	28–37
Total scores for coordination (Section B)			
*n*	21	6	27
Mean (SD)	29.5 (2.66)	31.2 (2.40)	29.9 (2.65)
Median	30.0	31.0	30.0
Range	25–33	27–34	25–34
Total scores for tone (Section C)			
*n*	21	6	27
Mean (SD)	18.6 (2.06)	16.0 (1.79)	18.0 (2.25)
Median	19.0	16.5	19.0
Range	13–21	13–18	13–21
Total scores for behaviour (Section D)			
*n*	21	6	27
Mean (SD)	12.3 (2.15)	14.7 (0.82)	12.9 (2.16)
Median	12.0	15.0	13.0
Range	8–15	13–15	8–15
Overall total scores (Section A + B + C + D)			
*n*	21	6	27
Mean (SD)	92.6 (7.12)	96.0 (5.40)	93.4 (6.83)
Median	95.0	97.0	96.0
Range	78–102	87–102	78–102

Median head circumference at 12 months of age, 45 cm, was within the WHO reference ranges at 1 year of age, i.e. 46.1 cm (43.5–48.6) for boys, and 44.9 cm (42.2–47.6) for girls [31].

## Discussion

The treatment of neonatal malaria and malaria in infants of body weight < 5 kg has been little studied, and current treatment recommendations are not based on the GRADE assessment of evidence [1]. A lack of therapies specifically formulated for young infants, with no antimalarials approved for use in patients weighing less than 4.5 kg, has led to empirical recommendations based on adjusted doses of available combination treatments or to the use of artesunate or quinine as monotherapy. Such approaches risk treatment being suboptimal. Simple dose adjustments of existing ACTs, for example, may risk exposing patients to artemether and DHA toxicity related to high artemether concentrations [22].

The current study is one of the first to evaluate antimalarial treatment in patients of < 5 kg body weight. We assessed a specifically designed fixed-ratio formulation of artemether–lumefantrine dispersible tablet developed for use in infants < 5 kg body weight, in the treatment of uncomplicated *P. falciparum* malaria. We utilized a PK bridging approach, supported by PBPK modelling [24], using exposure matching to extrapolate to the extensive efficacy and safety data of artemether–lumefantrine in older paediatric patients. This approach was taken given the potential for a limited number of patients to be recruited, particularly for Cohort 2, and is well-established and in line with ICH [32, 33], EMA [34], and FDA guidelines [35]. We based doses on body weight rather than body surface area, as adjustment by body weight is commonly used with antimalarials, and determination of body surface area may be challenging within endemic settings.

Neonates and infants < 5 kg with falciparum malaria represent a population with very specific physiological characteristics, and the very youngest patients, in particular, are difficult to recruit for clinical studies. Malaria in such patients may be asymptomatic, and the use of bed nets and antimalarial intermittent preventive therapy in pregnancy might be expected to reduce rates of congenital malaria, although this remains uncertain [36]. The extensive recruitment efforts in this study involved site assessments to confirm the presence of the eligible patient population in a resource-limited setting, necessitating upgrades to patient wards, labs, and pharmacy infrastructure. A decentralized recruitment network was established by involving peripheral health centres, supported by training and weekly communication. Early engagement with local authorities, including the national ethics committee, regulatory body, health zones, and hospital boards, was essential for allowing patient referrals from health centres to study sites. Community outreach was conducted through local health workers prior to recruitment start and during the trial. Despite these efforts over a 3-year period, while Cohort 1 patients were recruited as planned, only 6 of the planned 22 patients were recruited for Cohort 2.

Pharmacokinetics evaluations demonstrated that artemether and lumefantrine exposures in infants < 5 kg body weight treated with this optimized dose of 5 mg artemether and 60 mg lumefantrine were within the safe and efficacious range observed following treatment with the currently available dispersible tablet (20 and 120 mg) in older paediatric patients. The key PK assessments were artemether C_max_ (the primary endpoint of the study), which is associated with early parasite clearance, and lumefantrine C_168h_, which is an accepted marker for 28-day cure rate [37–39]. However, formal interpretation of artemether C_max_ was only possible for Cohort 1, as the sample size in Cohort 2 was too small. Lumefantrine C_max_ was also within the range observed in older paediatric patients treated with the currently available dispersible tablet. A PBPK model, reported separately [24] was also used to supplement the limited data from the study and provide additional support for the dose recommendations, particularly for patients < 5 kg and ≤ 28 days old. The model was developed to predict artemether C_max_ and lumefantrine C_168h_, and the predicted concentrations in infants and neonates (both ≤ 28 days and > 28 days of age) were within the safe and efficacious ranges observed in previous studies in older paediatric patients. As is well-characterized in other patient populations [21, 39], we noted high inter-individual variability in both artemether and lumefantrine concentrations in the study population.

At Day 29, the PCR-corrected ACPR was over 95% in both cohorts, and median PCT was similar to that in older paediatric patients treated with artemether–lumefantrine dispersible tablets (20 mg/120 mg) in a previous study [20]. The PCR-corrected ACPR rate was also evaluated using a Bayesian borrowing analysis, which also indicated a high probability of a response rate greater than 95%. These efficacy analyses were supportive of the PK analyses, also indicating that therapeutic concentrations had been reached. Treatment was well-tolerated; no deaths, SAEs, or treatment discontinuations occurred. Adverse events were consistent with those reported with artemether–lumefantrine dispersible tablets (20 mg/120 mg) in paediatric patients in a previous study [20]. Some changes from baseline in haemoglobin concentrations were observed; these were as expected in the patient population studied. Haemoglobin levels decline rapidly after birth, reaching their lowest point 9–11 weeks after delivery, then increasing [40]. In addition, malaria in neonates and young infants is known to cause anaemia, even at low parasite densities [41]. Liver function test abnormalities occurred in a proportion of patients. Elevations in liver function tests occur commonly in patients with malaria [42] as the liver is a target organ for malaria parasites, and increased liver function test values are listed as a common adverse effect for artemether–lumefantrine [43].

Long-term follow-up when patients reached 12 months of age showed neurodevelopment scores and head circumference within the normal ranges.

Limitations of the study included the open-label, uncontrolled design. A placebo-controlled design would clearly have been unethical, and given that there are no approved treatments for patients in this age group, it was not possible to identify an active control. However, as the pharmacokinetic and efficacy parameters used are all objective measurements that are well-established in studies of malaria, the design should not have affected these outcomes, and uncontrolled designs are commonly used in studies in paediatric patients with malaria.

Another limitation of the study was the small sample size, which is a product of the difficulties in recruitment of the very young patients in Cohort 2, particularly patients less than 15 days of age, of whom only a single patient was recruited. However, the sample size was adequate for the PK bridging approach and to assess the primary endpoint in the overall study population and Cohort 1, and a PBPK model was used to supplement the observed data in Cohort 2. While the study was not powered for efficacy assessments, a Bayesian analysis was undertaken to further explore the efficacy data. One final limitation relates to safety evaluation, where due to the young age of the patients they would have been unable to report subjective symptoms, so the safety profile characterized in the study is essentially limited to objective signs as observed by medical staff or the patients’ parents or guardians. Given the difficulties of conducting the study, alternative methods of obtaining data, such as keeping a neonatal registry might be of value in the future, in terms of monitoring malaria cases in this patient population and their response to treatment.

The 1:12 artemether–lumefantrine dispersible tablet assessed in this study has been approved by Swissmedic as the first malaria medicine for newborns and young infants (2– < 5 kg body weight). Eight African countries also participated in the assessment and are now expected to complete their assessment under the Swiss agency’s Marketing Authorization for Global Health Products procedure. Novartis plans to make the infant-friendly treatment available to increase access in areas where malaria is endemic.

## Conclusions

In this study, artemether and lumefantrine exposures, efficacy, and safety following treatment of patients of < 5 kg body weight with *P. falciparum* malaria with a dispersible artemether–lumefantrine tablet with an optimized dose for such patients (each dose 5 mg artemether and 60 mg lumefantrine) were consistent with those in patients 5– < 15 kg treated with the currently available dispersible tablet formulation (20 mg artemether and 120 mg lumefantrine per tablet) in previous studies. The study provides evidence to support the use of this optimized dose dispersible tablet for treatment of patients < 5 kg with *P. falciparum* malaria.

## Supplementary Information

Supplementary Material 1. Full study entry criteria.

Supplementary Material 2. Pharmacokinetic data from Study COA566B2303 (NCT00386763).

Supplementary Material 3. Bayesian borrowing analysis methodology.

## Data Availability

The sponsor of this study is committed to sharing with qualified external researchers access to patient-level data and supporting clinical documents from eligible studies. These requests are reviewed and approved by an independent review panel on the basis of scientific merit. All data provided are anonymized to respect the privacy of patients who have participated in the trial in line with applicable laws and regulations. This trial data availability is according to the criteria and process described on [http://www.clinicalstudydatarequest.com].
