# Supplementary material for: Pharmacokinetics, safety and efficacy of an optimized dose of artemether–lumefantrine in the treatment of acute uncomplicated Plasmodium falciparum malaria in neonates and infants of less than 5 kg body weight: a multicentre, open-label, single-arm phase 2/3 study (CALINA)
Source: Trop Med Health. 2025 Nov 6;53:151. doi: 10.1186/s41182-025-00828-z (PMC12590907; doi:10.1186/s41182-025-00828-z)
Supplement: Supplementary file 1 — Supplementary Material 1. Full study entry criteria. [file 41182_2025_828_MOESM1_ESM.pdf]

Pharmacokinetics, safety, and efficacy of an optimized dose of artemether-lumefantrine in the treatment of acute uncomplicated *Plasmodium falciparum* malaria in neonates and infants of less than 5 kg body weight: a multicenter, open-label, single arm Phase 2/3 study (CALINA)

Gildas Wounounou et al

Additional file 1: Full study entry criteria

Inclusion criteria

Patients eligible for inclusion in this study fulfilled all of the following inclusion criteria.

- Male or female neonates/infants
- Body weight <5 kg but ≥2 kg
- In Cohort 1, infants aged >28 days; in Cohort 2, neonates aged 1 to ≤28 days (3 sub-groups: 1-7 days; 8-14 days; 15-28 days)
- Microscopically confirmed diagnosis of *P. falciparum* malaria (or mixed infections):
    - In Cohort 1 of ≥500 and <100000 parasites/μL asexual *P. falciparum* parasitemia
    - In Cohort 2 of ≥100 and <100000 parasites/μL asexual *P. falciparum* parasitemia
    - either congenital or neonatal
    - either symptomatic or asymptomatic

Exclusion criteria

Patients fulfilling any of the following exclusion criteria were not eligible for inclusion in this study.

- Head circumference <-2 SD z-score in cm following WHO age and sex-specific reference curves (suspicion of microcephaly)
- Presence of severe malaria (according to WHO 2015 definition)
- HIV status:
    - In Cohort 1, patient's or patient's mother's current treatment with ARV
    - In Cohort 2, mother's known HIV positive status at patient's birth or mother's current treatment with ARV
- Severe malnutrition (BMI <70% of median normalized WHO reference weight)
- Presence of the following signs of a critical condition: apnea-bradycardia, sustained bradycardia, tachycardia, desaturation, hypotension, hypothermia; or other severely deteriorated general condition (based on IMCI criteria in sick infants) (WHO 2005)
- Presence of any clinically significant neurological condition:
    - Any episode of convulsion during the present illness (in keeping with the IMCI list of general danger signs)
    - Known neurological disorders (e.g. chronic seizure disorders, cerebral palsy)
- Presence of clinically significant abnormality of the hepatic and renal systems
- Patients unable to swallow or whose drinking is impaired
- Known hypersensitivity of either the patient or patient's parent to artemether, lumefantrine, any of the excipients of Coartem®/Riamet® Dispersible tablet, or to drugs of similar chemical classes
- History of malabsorption or previous gastrointestinal surgery, or history of radiation therapy that could affect drug absorption or metabolism, or any other disorder or history of a condition that could interfere with drug absorption, distribution, metabolism, or excretion
- Known family history of congenital prolongation of the QTc interval or sudden death or with any other clinical condition known to be associated with prolongation of the QTc interval such as history of symptomatic cardiac arrhythmias, with clinically relevant bradycardia or with severe cardiac disease
- Disturbances of electrolyte balance (e.g. hypokalemia or hypomagnesemia)

- Presence of any age-adjusted clinically or hematologically relevant laboratory and blood chemistry abnormalities (including hemoglobin <7 g/dL)
- Patients who received any antimalarial drug, including antibiotics with antimalarial activity, within 14 days of trial start, or any other prohibited drug
- Patients who received an investigational drug within 5 half-lives of enrollment or participated in an investigational study or within 30 days, whichever is longer

Where information was either not available or insufficient (e.g. from medical history, charts, etc.) to assess whether a patient met a specific exclusion criterion, the study site team used its best medical judgement to make an inclusion/exclusion decision for the patient.
