# Supplementary material for: Pharmacokinetics, safety and efficacy of an optimized dose of artemether–lumefantrine in the treatment of acute uncomplicated Plasmodium falciparum malaria in neonates and infants of less than 5 kg body weight: a multicentre, open-label, single-arm phase 2/3 study (CALINA)
Source: Trop Med Health. 2025 Nov 6;53:151. doi: 10.1186/s41182-025-00828-z (PMC12590907; doi:10.1186/s41182-025-00828-z)
Supplement: Supplementary file 2 — Supplementary Material 2. Pharmacokinetic data from Study COA566B2303 (NCT00386763). [file 41182_2025_828_MOESM2_ESM.pdf]

Pharmacokinetics, safety, and efficacy of an optimized dose of artemether-lumefantrine in the treatment of acute uncomplicated *Plasmodium falciparum* malaria in neonates and infants of less than 5 kg body weight: a multicenter, open-label, single arm Phase 2/3 study (CALINA)

Gildas Wounounou et al

Additional file 2: Pharmacokinetic data from Study COA566B2303 (NCT00386763)

Parameter	n	Mean +/- SD (CV%)	Median (Range)	Geometric mean (90% CI)
C_{max} artemether (ng/mL)	52	195.660 +/- 203.717 (104.1)	158.000 (0.000, 932.000)	101 (73.4, 140)
C_{max} DHA (ng/mL)	52	61.997 +/- 64.844 (104.6)	40.900 (0.000, 286.000)	31.7 (23.2, 43.5)
C_{max} Lumefantrine (µg/mL)	102	5.097 +/- 3.669 (72.0)	4.035 (0.069, 16.000)	3.85 (3.36, 4.42)
Lumefantrine C_{168h} (µg/mL)	27	0.296 +/- 0.237 (80.2)	0.257 (0.000, 1.070)	0.212 (0.156, 0.287)

Data shown are for patients of body weight 5- <15 kg treated with the artemether-lumefantrine dispersible tablet. Study design and some pharmacokinetic data presented in Abdullah et al 2008 and Djimde et al 2011, respectively.
