# Supplementary material for: Pharmacokinetics, safety and efficacy of an optimized dose of artemether–lumefantrine in the treatment of acute uncomplicated Plasmodium falciparum malaria in neonates and infants of less than 5 kg body weight: a multicentre, open-label, single-arm phase 2/3 study (CALINA)
Source: Trop Med Health. 2025 Nov 6;53:151. doi: 10.1186/s41182-025-00828-z (PMC12590907; doi:10.1186/s41182-025-00828-z)
Supplement: Supplementary file 3 — Supplementary Material 3. Bayesian borrowing analysis methodology. [file 41182_2025_828_MOESM3_ESM.pdf]

Pharmacokinetics, safety, and efficacy of an optimized dose of artemether-lumefantrine in the treatment of acute uncomplicated *Plasmodium falciparum* malaria in neonates and infants of less than 5 kg body weight: a multicenter, open-label, single arm Phase 2/3 study (CALINA)

Gildas Wounounou et al

Additional file 3: Bayesian borrowing analysis methodology

1 Historical studies included in analysis

The following Novartis internal studies in patients with uncomplicated *P. falciparum* malaria were included in the analysis:

- COA566A025 (Van Vugt et al 1999): a randomized, double-blind, parallel group trial comparing efficacy, safety and pharmacokinetics of the standard schedule (4x4 tablets over 48 hours) with two higher dose schedules of artemether-lumefantrine in the treatment of acute *Plasmodium falciparum* malaria in adults and children in Thailand.
- COA566A026 (Van Vugt et al 2000): a randomized trial confirming efficacy and safety of the high dose regimen of artemether-lumefantrine in comparison with mefloquine/artesunate in the treatment of acute *Plasmodium falciparum* malaria in adults and children in Thailand.
- COA566A2403 (Falade et al 2005): an open label, multicenter study for the evaluation of safety and efficacy of artemether-lumefantrine tablets (6-dose regimen) in African infants and children in the treatment of acute uncomplicated falciparum malaria.
- COA566B2303 (Abdullah et al 2008): a randomized, investigator-blinded, multicenter, parallel-group study to compare efficacy, safety, and tolerability of artemether-lumefantrine dispersible tablet formulation vs. artemether-lumefantrine 6-dose crushed tablet in the treatment of acute uncomplicated Plasmodium falciparum malaria in infants and children.
- COA566B2306 (Tiono et al 2015): an open-label, single-arm study to evaluate the efficacy, safety, and PK of artemether-lumefantrine dispersible tablet in the treatment of acute uncomplicated Plasmodium falciparum malaria in infants <5 kg body weight.
- KAF156A2202 Part B (Ogutu et al 2023): a Phase 2 interventional, multicenter, randomized open label study to determine the effective and tolerable dose of ganaplacide plus and lumefantrine solid dispersion formulation in combination, given once daily for 1, 2 and 3 days to adults and children with uncomplicated Plasmodium falciparum malaria. In this study artemether-lumefantrine was used as a control arm. Only the artemether-lumefantrine arm and related results will be used from this study.

These studies enrolled children <12 years and analyzed PCR corrected ACPR at Day 29. The analyses were based on results from the evaluable or PPS population of the 6 historical artemether-lumefantrine studies mentioned above (Table 1).

Table 1 Novartis historical pediatric studies

Study (Year of conduct)	Artemether-lumefantrine formulation	No. of patients treated with artemether-lumefantrine (ITT)	Age Range (Years)	PCR corrected ACPR@D29 n/N (%)*	Source
A025, Age <12Y (1996-97)	Standard tablet	16	3-11	11/11 (100%)	Post hoc analysis
A026, Age <12Y (1998-99)	Standard tablet	19	2-11	16/17 (94.1%)	Post hoc analysis
A2403 (2001-05)	Standard tablet	310	0.2-9.9	289/299 (96.7%)	Makanga and Krudsood (2008)
B2303 (2006-07)	Standard tablet	452	0.0-12.0	403/409 (98.5%)	Abdullah et al 2008
	Dispersible tablet	447	0.0-12.0	394/403 (97.8%)	
B2306 (2012-14)	Dispersible tablet	20	0.1-0.6	16/16 (100%)	Tiono et al 2015
KAF156A2202 Part B (2020-21)	Dispersible tablet	24	2-11	21/22 (95.5%)	Ogutu et al 2024

* Based on the primary analysis population in each study.

The results for PCR-corrected ACPR at Day 29 from each study are used as the source data for analysis using the R package RBesT.

2 Statistical methods

The objective of proposed Bayesian approaches for borrowing Efficacy

As the historical data included studies which varied in their time of conduct and in the artemether-lumefantrine formulation used, between-study heterogeneity was accounted for through the use of a random effects meta-analysis. In addition, the analysis was performed in two steps aiming to first qualify the relevance of the historical data and then perform the borrowing, with the overall objectives:

- To confirm that PCR corrected ACPR at Day 29 observed in study B2307 is in alignment with PCR corrected ACPR at Day 29 in historical artemether-lumefantrine studies.
- To provide a more precise estimate of PCR corrected ACPR at Day 29 by leveraging available information from historical artemether-lumefantrine studies through Bayesian borrowing approach.

Bayesian borrowing approach.

Steps undertaken:

1) Bayesian Meta-Analytic-Predictive (MAP) analysis was performed (Neuenschwander et al 2010) to synthesize the historical data as a MAP prior (P_{MAP}). The MAP analysis was performed using the R package RBesT. The priors used for the MAP analysis are a Half Normal distribution for tau (between study standard deviation) with a standard deviation of 1 (large between trial variation, Friede et al 2016) and a normal distribution for the overall intercept beta with a mean of 0 and a standard deviation of 2. The response rates for the trials and MAP are displayed in a forest plot. The resulting MCMC sample for P_{MAP} are summarized by a mixture of 3 component beta distributions which are fitted using an expectation-maximization algorithm implemented in RBesT. The median response rate and 95% credible interval are displayed.

2) The 2-sided 95% predictive interval for the number of responders for given number of patients in the current study was calculated assuming that the response rate follows P_{MAP}. If the observed number of responders is equal to or greater than the lower limit of the predictive interval, the observed response rate is considered to be in alignment with the historical artemether-lumefantrine response rate. This step served as a check of consistency of current study data and historical data. The given number of patients for predicting was primarily based on the number of patients in the Per Protocol Set (PPS) in Study B2307 (Cohort 1 alone, Cohort 2 alone, and Cohorts 1 and 2 pooled). Sensitivity analyses was performed using the full analysis set (FAS) as the patient with recrudescence at Day 29 was excluded from the PPS due to administration of prohibited medication.
3) The P_{MAP} was made robust using an uninformative beta (1/3, 1/3) with weight of 0.2. Beta (1/3, 1/3) is equivalent to information with 2/3 patient with 50% response rate and is also known as a neutral prior, since the resulting posterior distribution has a median which equals the respective maximum likelihood estimate (Kerman 2011). A weight of 20% for the uninformative prior component represents our prior assumption that with 20% probability the new data will not be exchangeable to the historical data. The robustified P_{MAP} was used as the prior to the observed number of responders. The posterior distribution for the response rate is summarized using:
   a) The 50th percentile and 95% credible interval (CrI) for the response rate,
   b) The posterior probability of response rate >90%,
   c) The posterior probability of response rate >95%.
4) A sensitivity analysis was performed by varying the weight for the uninformative prior component of the beta (1/3, 1/3) from 0 to 1.0 in steps of 0.1. The goal was to identify a tipping point weight for which the probability to exceed a response rate of 90% drops below 0.975.

Note: Since the weighting of 0.2 in Step 3) is one scenario for the sensitivity analysis in Step 4), the results in Step 3) were embedded in the results of Step 4).

The above steps were conducted for the analysis in Cohort 1 and pooled (Cohorts 1 & 2) for the study COA566B2307. For Cohort 2 alone in COA566B2307, Cohort 1 data were added as a new study in Step 1 but assigned a separate stratum for which an additional heterogeneity parameter tau is introduced with a prior reflecting small between trial heterogeneity. For this a Half Normal with standard deviation ¼ was used and the P_{MAP} was derived within this additional stratum.

3 MAP priors

Five patients in Cohort 1 were excluded from the PPS because they took prohibited medications, one of them experienced parasite recrudescence. All patients in Cohort 2 are included in the PPS and none of them experienced parasite recrudescence. Therefore, the PCR corrected ACPR at Day 29 was analyzed also using FAS in addition to the pre-planned PPS for Cohort 1 alone and Cohorts 1 and 2 pooled.

Since Cohort 1 was included as a new stratum in the MAP prior for the analysis of Cohort 2 alone, the MAP prior for Cohort 2 alone was performed in 2 ways, one using Cohort 1 PPS and the other using Cohort 1 FAS.

The historical artemether-lumefantrine studies listed in Table 2 were synthesized into a MAP prior for the analysis of Cohort 1 alone and Cohorts 1 and 2 pooled. The MAP prior is presented in Section 3.1.

The MAP priors and respective medians for the different cohorts and analysis sets were very similar, see details below.

- For Cohort 1, Cohort 1 and 2 pooled in FAS or PPS: 97.6% (95% CrI: 91.7% to 99.3%)
- For Cohort 2 using Cohort 1 PPS as part of historical data: 97.7% (95% CrI: 90.8% to 99.3%)
- For Cohort 2 using Cohort 1 FAS as part of historical data: 97.5% (95% CrI: 90.9% to 99.2%)

MAP prior for Cohort 1 alone and Cohorts 1 and 2 pooled

There were a total of 1177 patients and 1150 responders (PCR corrected ACPR at Day 29) in Table 1. The overall response rate was 97.7% (1150/1177).

The historical artemether-lumefantrine studies and MAP prior are plotted in Figure 1. The resulting MCMC sample for MAP prior was summarized by a mixture of 3 component beta distributions displayed in Table 2. The median and 95% creditable limit for the mixture beta distribution was 97.6% (91.7% to 99.3%).

Figure 1. Forest plot of Novartis historical artemether-lumefantrine studies and MAP prior for Cohort 1 alone and Cohorts 1 and 2 pooled

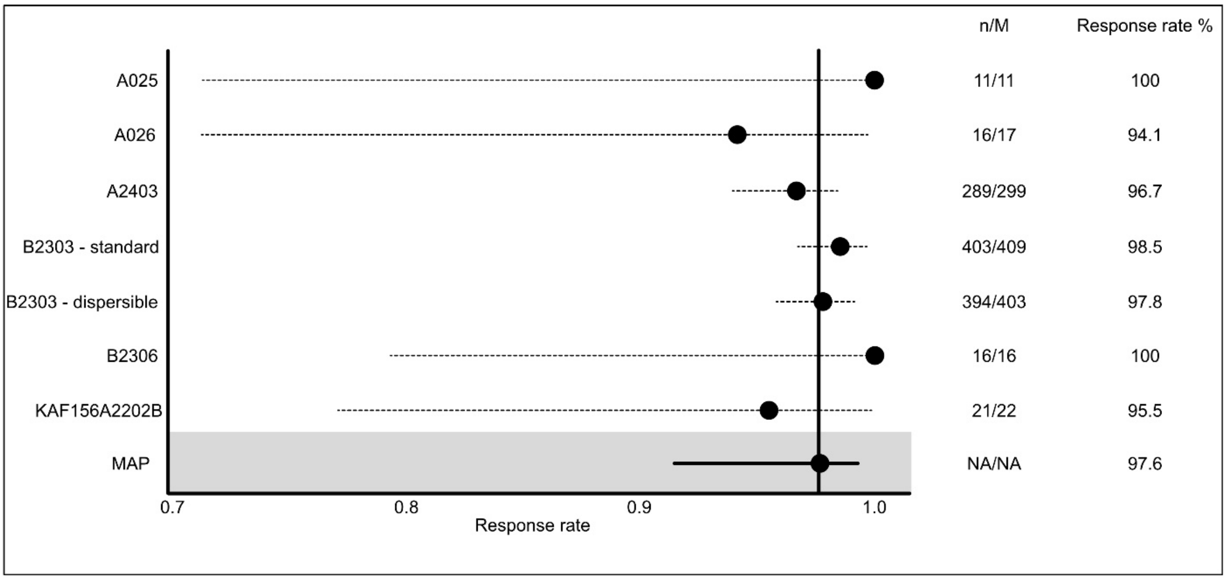

Table 2 Mixture beta components of the MAP prior for Cohort 1 alone and Cohorts 1 and 2 pooled

Beta component	Component 1	Component 2	Component 3
Weight	0.6268980	0.3394117	0.0336903
a	503.3551053	64.5341867	9.7507386
b	12.0832287	2.0921125	1.1115405

This mixture beta distribution was used for the estimate of PCR corrected ACPR at Day 29 for Cohort 1 alone and Cohorts 1 and 2 pooled based on the PPS and the FAS. The 2-sided 95%

predictive interval for the number of responders are displayed in Table 3. The observed number of responders is greater than the lower limit of 2-sided 95% predictive limit for all 4 methods, which confirm that PCR corrected ACPR at Day 29 observed in this study was in alignment with PCR corrected ACPR at Day 29 in historical artemether-lumefantrine studies.

Table 3 Two-sided 95% predictive limit for number of responders in Cohort 1 alone and Cohorts 1 and 2 pooled

Cohort	Analysis set	Number of patients	Two-sided 95% predictive limit for number of responders	Observed number of responders
Cohort 1	PPS	17	15, 17	17
Cohort 1	FAS	22	19, 22	21
Cohorts 1 and 2 pooled	PPS	23	20, 23	23
Cohorts 1 and 2 pooled	FAS	28	25, 28	27

4. Results

Posterior results for Cohorts 1 and 2 pooled based on FAS

Table 4 presents the posterior results for PCR corrected ACPR rate at Day 29 for Cohorts 1 and 2 pooled based on FAS (27/28 [96.4%]) by varying borrowing information in steps of 0.1. The median and 95% credible interval were plotted in Figure 2 and the probability of response rate>90% and probability of response rate>95% were plotted in Figure 3.

As the amount of borrowing (weight for MAP prior) increased from 0 to 1, the following were observed:

- The resulting (robustified) prior changes in that the median moved from 50% without any historical data to 97.6% when using the MAP prior on its own. The lower limit of 95% credible interval for the resulting prior started with 0.0% and increased slightly until the amount of borrowing reached 0.9 (90%), and then jumped to 91.7% when the amount of borrowing was 1. The very wide 95% CrI indicated some uncertainty for the resulting prior when the amount of borrowing was ≤0.9.
- The median of the posterior distribution increased slightly from 96.4% to 97.5% but stabilized at about 97.5% when the amount of borrowing reached 0.3 (see Figure 2).
- The width of 95% credible interval for the posterior distribution decreased, indicating more precise estimating. The width decrease was mainly driven by the increase of the lower limit of 95% credible interval. The incremental increase of the lower limit of 95% credible interval became smaller as the amount of borrowing increased (Figure 2).
- Posterior probability of response rate >90% increased and reached 0.975 when the amount of borrowing was 0.4 or higher:
    - o The tipping weight that the probability of rate >90% changed from <0.975 to first >=0.975 was 0.4;
    - o The posterior probability of response rate >90% was 0.9926 for the pre-specified borrowing of 0.8.
- Posterior probability of response rate >95% increased and reached about 0.8 or higher when the amount of borrowing was 0.2 or higher (Figure 3).

Table 4 Posterior results for PCR corrected ACPR rate at Day 29 for Cohorts 1 and 2 pooled by varying borrowing information based on FAS

Weight for MAP prior	Weight for non-informative beta (1/3, 1/3)	Prior distribution		Posterior distribution (after borrowing)		Probability (Rate>90%)	Probability (Rate>95%)
		Median (%)	95% CrI	Median (%)	95% CrI		
0	1	50	0, 100	96.4	85.3, 99.7	0.9034	0.639
0.1	0.9	61.5	0, 100	97.2	86.9, 99.6	0.9391	0.754
0.2	0.8	74.6	0, 100	97.3	88.2, 99.5	0.9577	0.814
0.3	0.7	87.4	0, 100	97.4	89.3, 99.4	0.9691	0.851
0.4*	0.6	94.7	0, 100	97.5	90.3, 99.3	0.9769	0.875
0.5	0.5	96.5	0.1, 99.9	97.5	91.1, 99.2	0.9824	0.893
0.6	0.4	97	0.1, 99.9	97.5	91.8, 99.1	0.9866	0.907
0.7	0.3	97.3	0.3, 99.8	97.5	92.4, 99.1	0.9899	0.917
0.8#	0.2	97.4	1.1, 99.6	97.5	92.8, 99	0.9926	0.926
0.9	0.1	97.5	8.3, 99.4	97.5	93.2, 99	0.9947	0.933
1	0	97.6	91.7, 99.3	97.5	93.5, 99.0	0.9965	0.939

CrI: Credible interval
#: Pre-specified borrowing
*Tipping weight that the probability of rate>90% changes from <0.975 to first >=0.975.

Figure 2. Posterior median and 95% credible interval (CrI) for PCR corrected ACPR rate at Day 29 for Cohorts 1 and 2 pooled by varying borrowing information based on FAS

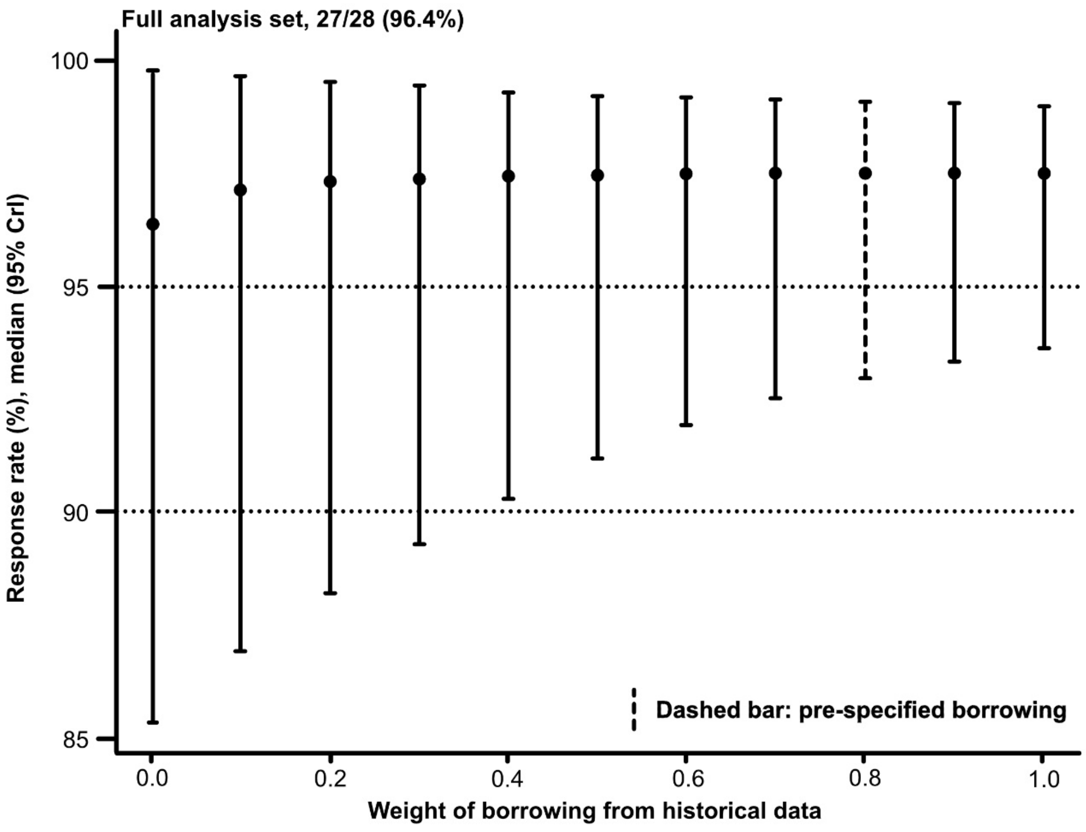

Figure 3. Posterior probabilities for PCR corrected ACPR rate at Day 29 greater than 90% and 95% for Cohorts 1 and 2 pooled by varying borrowing information based on FAS

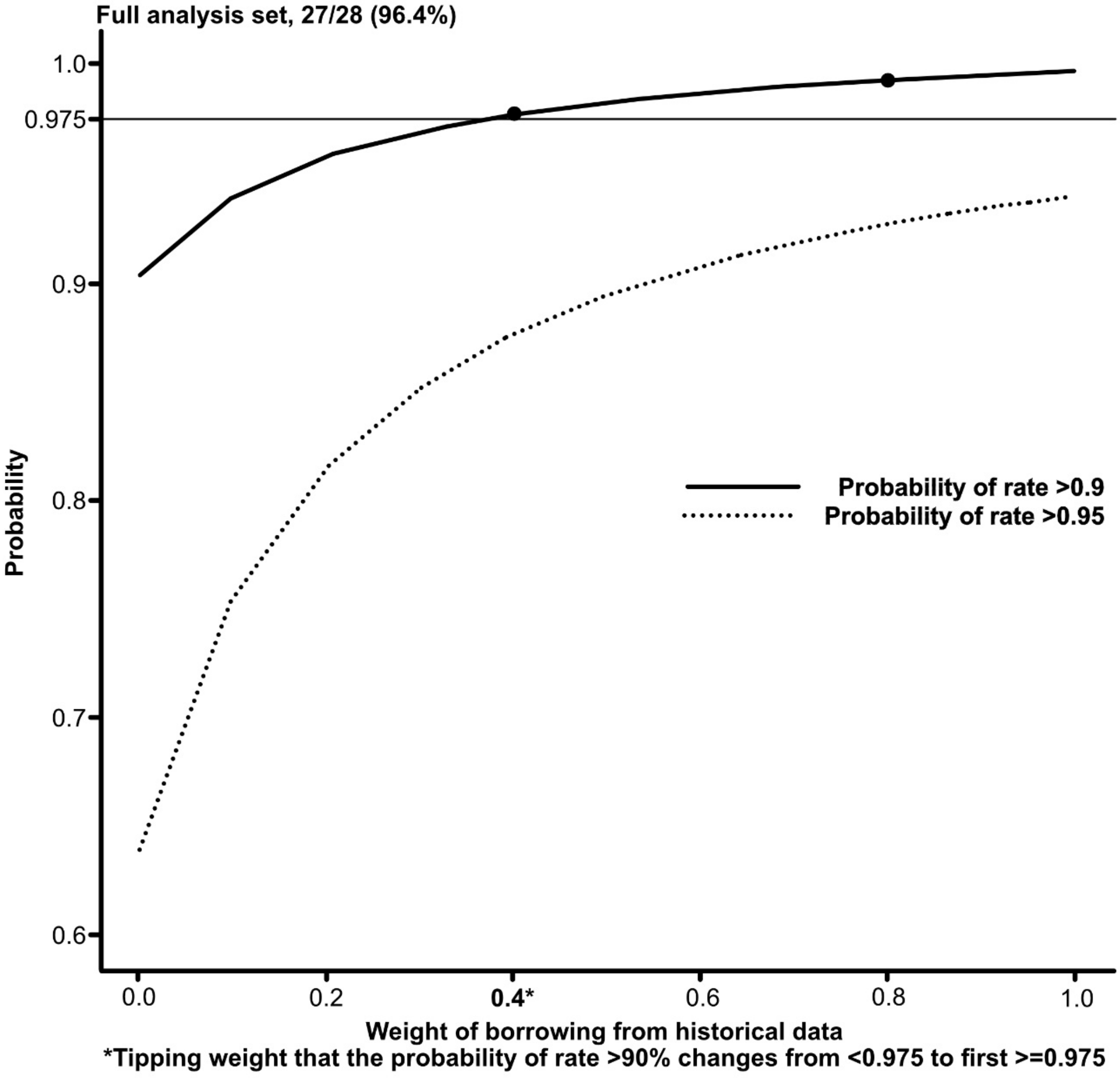

5 References

Friede T, Rover C, Wandel S, and Neuenschwander B (2016). Meta-analysis of few small studies in orphan diseases", Research Synthesis Methods, DOI: 10.1002/jrsm.1217.

Kerman J. (2011) Neutral noninformative and informative conjugate beta and gamma prior distributions. Electronic Journal of Statistics, Vol. 5, P1450-1470.

Neuenschwander B, Capkun-Niggli G, Branson M, and Spiegelhalter D (2010) Summarizing historical information on controls in clinical trials. Clinical Trials, Vol. 7, P5-18.

van Vugt M, Looareesuwan S, Wilairatana P, et al (2000). Artemether-lumefantrine for the treatment of multidrug-resistant falciparum malaria. Trans R Soc Trop Med Hyg;94(5):545-8

van Vugt M, Wilairatana P, Gemperli B, et al (1999). Efficacy of six doses of artemether-lumefantrine (benflumetol) in multidrug-resistant Plasmodium falciparum malaria. Am J Trop Med Hyg. 1999 Jun;60(6):936-42.

Falade C, Makanga M, Premji Z, Ortmann CE, Stockmeyer M, de Palacios PI. Efficacy and safety of artemether-lumefantrine (Coartem) tablets (six-dose regimen) in African infants and children with acute, uncomplicated falciparum malaria. Trans R Soc Trop Med Hyg. 2005 Jun;99(6):459-67.

Tiono AB, Tinto H, Alao MJ, Meremikwu M, Tshefu A, Ogutu B, et al. Increased systemic exposures of artemether and dihydroartemisinin in infants under 5kg with uncomplicated Plasmodium falciparum malaria treated with artemether-lumefantrine (Coartem®). Malar J. 2015;14:157.

Ogutu B, Yeka A, Kusemererwa S, et al (2023). Ganaplacide (KAF156) plus lumefantrine solid dispersion formulation combination for uncomplicated Plasmodium falciparum malaria: an open-label, multicentre, parallel-group, randomised, controlled, phase 2 trial. Lancet Infect Dis;23(9):1051-1061.

Makanga M, Krudsood S (2009). The clinical efficacy of artemether/lumefantrine (Coartem®). Malaria J; 8(Suppl 1):S5.
